# Cell-penetrating peptides, targeting the regulation of store-operated channels, slow decay of the progesterone-induced [Ca^2+^]_i_ signal in human sperm

**DOI:** 10.1093/molehr/gav019

**Published:** 2015-04-16

**Authors:** Jennifer Morris, Sarah Jones, John Howl, Monika Lukanowska, Linda Lefievre, Stephen Publicover

**Affiliations:** 1 School of Biosciences, University of Birmingham, Edgbaston, Birmingham B15 2TT, UK; 2 Molecular Pharmacology Research Group, Research Institute in Healthcare Science, University of Wolverhampton, Wolverhampton WV1 1LY, UK; 3 The Medical School, University of Birmingham, Edgbaston, Birmingham B15 2TT, UK

**Keywords:** spermatozoa, cell-penetrating peptide, stromal interaction molecule 1, store-operated channels

## Abstract

Previous work has provided evidence for involvement of store-operated channels (SOCs) in [Ca^2+^]_i_ signalling of human sperm, including a contribution to the transient [Ca^2+^]_i_ elevation that occurs upon activation of CatSper, a sperm-specific cation channel localized to the flagellum, by progesterone. To further investigate the potential involvement of SOCs in the generation of [Ca^2+^]_i_ signals in human sperm, we have used cell-penetrating peptides containing the important basic sequence KIKKK, part of the STIM–Orai activating region/CRAC activating domain (SOAR/CAD) of the regulatory protein stromal interaction molecule 1. SOAR/CAD plays a key role in controlling the opening of SOCs, which occurs upon mobilization of stored Ca^2+^. Resting [Ca^2+^]_i_ temporarily decreased upon application of KIKKK peptide (3–4 min), but scrambled KIKKK peptide had a similar effect, indicating that this action was not sequence-specific. However, in cells pretreated with KIKKK, the transient [Ca^2+^]_i_ elevation induced by stimulation with progesterone decayed significantly more slowly than in parallel controls and in cells pretreated with scrambled KIKKK peptide. Examination of single-cell responses showed that this effect was due, at least in part, to an increase in the proportion of cells in which the initial transient was maintained for an extended period, lasting up to 10 min in a subpopulation of cells. We hypothesize that SOCs contribute to the progesterone-induced [Ca^2+^]_i_ transient, and that interference with the regulatory mechanisms of SOC delays their closure, causing a prolongation of the [Ca^2+^]_i_ transient.

## Introduction

[Ca^2+^]_i_ plays a central role in the control of sperm function. Maturation of the ejaculated sperm to acquire competence to fertilize (capacitation), the regulation of motility pattern (behaviour and chemotaxis) and control of the secretion of the acrosomal vesicle (acrosome reaction), which releases hydrolytic enzymes and exposes proteins required for fertilization at the sperm surface, are all controlled separately through [Ca^2+^]_i_ signals. Ca^2+^ channels and Ca^2+^ stores are present in mammalian sperm (Publicover *et al.*, 2007; Costello *et al.*, 2009; Darszon *et al.*, 2011) and both play important roles in sperm function. CatSper, a sperm-specific cation channel localized to the flagellum, is the primary Ca^2+^-influx channel in mammalian sperm, is central to the regulation of [Ca^2+^]_i_ and experiments in CatSper-null mice have shown that the channels play a key role in both regulation of motility and the early phase of zona pellucida-induced acrosome [Ca^2+^]_i_ signalling (Carlson *et al.*, 2003; Xia and Ren, 2009). Intriguingly, ‘late’ [Ca^2+^]_i_ responses persisted in the mutant mice and the zona pellucida-induced acrosome reaction was not inhibited (Xia and Ren, 2009). Ca^2+^ stores are present both within the acrosome and at the sperm neck region (Costello *et al.*, 2009). Release of Ca^2+^ stored in the acrosome is required for completion of the acrosome reaction, and the Ca^2+^ stored at the sperm neck plays a key role in regulation of motility (Costello *et al.*, 2009; Alasmari *et al.*, 2013).

The characteristics of [Ca^2+^]_i_ signals generated by store mobilization in human sperm are consistent with the occurrence of capacitative Ca^2+^ entry (CCE) mediated by store-operated channels (SOCs) (Blackmore, 1993; Park *et al.*, 2011; Lefievre *et al.*, 2012). SOCs are activated by interaction with stromal interaction molecule (STIM), a sensor molecule present in the membrane of the Ca^2+^ store that detects depletion of stored Ca^2+^. Upon store mobilization, STIM redistributes, moving to a position adjacent to the plasma membrane and forming ‘puncta’, where it interacts with the channel proteins (Orai and possibly members of the transient receptor potential canonical [TRPC] family), causing SOCs to open (Cahalan, 2009). STIM1, Orai proteins and TRPC proteins have all been detected in human sperm (Castellano *et al.*, 2003; Darszon *et al.*, 2012; Lefievre *et al.*, 2012). STIM1 is localized primarily to the neck region/midpiece and the acrosome, the areas where Ca^2+^ stores are present (Lefievre *et al.*, 2012).

Progesterone, a product of the cumulus cells which surround the oocyte, is the best characterized agonist of Ca^2+^ signalling in human sperm. Progesterone directly activates CatSper channels, causing an immediate and transient Ca^2+^ elevation which peaks within 30 s and decays within 1–2 min (Blackmore *et al.*, 1990; Lishko *et al.*, 2011; Strunker *et al.*, 2011). This is followed by a prolonged [Ca^2+^]_i_ plateau. In a subpopulation of cells, this plateau phase includes repeated [Ca^2+^]_i_ elevations (oscillations; Harper *et al.*, 2004; Kirkman-Brown *et al.*, 2004; Aitken and McLaughlin, 2007; Sanchez-Cardenas *et al.*, 2014), which are typically irregular/chaotic but are ‘organized’ by ryanodine and by caffeine, consistent with Ca^2+^-induced Ca^2+^ release (CICR) from the store at the sperm neck (Harper *et al.*, 2004). These oscillations modify flagellar beat (Harper *et al.*, 2004) and may suppress the acrosome reaction (Sanchez-Cardenas *et al.*, 2014). We recently showed that a low concentration of 2-aminoethoxydiphenyl borate (2-APB; 5 µM), which potentiates SOC activity by promoting interaction with STIM, enhanced the progesterone transient at the sperm neck (but not the flagellum; Lefievre *et al.*, 2012). The [Ca^2+^]_i_ plateau phase was not enhanced by 2-APB pretreatment, but application of 2-APB during the plateau caused generation of irregular [Ca^2+^]_i_ noise and spikes. Patch-clamp experiments showed that 2-APB inhibited currents through CatSper channels. These observations suggest that store emptying by CICR and consequent activation of SOCs amplify the initial CatSper-mediated [Ca^2+^]_i_ transient and generate [Ca^2+^]_i_ oscillations.

Use of cell-penetrating peptides (CPPs) to traffic targeted peptides and small proteins across the plasma membrane offers an alternative potentially more specific technique than pharmacological intervention. Rab3A and GST, both ∼25 kDa, are delivered into sperm when fused to a polyarginine CPP (RRRQRRKRRRQ) at the C terminus (Lopez *et al.*, 2007) and the translocation of exogenous Rab3A is sufficient to trigger acrosomal exocytosis. We recently investigated the ability of a number of fluorescently labelled CPPs to translocate into sperm, identifying effective CPPs (Jones *et al.*, 2013). We have now exploited this technique to further investigate functional expression of SOCs in human sperm and their potential participation in the [Ca^2+^]_i_ response to progesterone by using CPPs to target functioning of SOCs in sperm. Though there are competing models for regulation of SOC activity, the importance of a region within the STIM molecule termed CAD (CRAC-activating domain) or SOAR (STIM–Orai activating region) is clear (Yuan *et al.*, 2009). Whereas the full cytoplasmic segment of STIM1 is a poor activator of Orai1, the CAD/SOAR domain is more effective (Korzeniowski *et al.*, 2010). This suggests that in the full length form, intramolecular interactions maintain STIM inactive until store depletion occurs. Unfolding of STIM then allows interaction with Orai. SOAR includes an internal basic patch which may interact with an acidic region on Orai to stabilize such folding (Korzeniowski *et al.*, 2010; Kim and Muallem, 2011; Soboloff *et al.*, 2011). To further investigate the potential involvement of SOCs in the generation of [Ca^2+^]_i_ signals in human sperm, we have used a CPP (KIKKK) that mimics the basic patch on the SOAR region of STIM. KIKKK may act directly to stimulate CCE by binding Orai or may interact with the acidic patch on STIM, thus interfering with the refolding that would normally render STIM quiescent upon store refilling.

## Methods

### Preparation and capacitation of spermatozoa

Healthy donors were recruited in accordance with the Human Fertilisation and Embryology Authority Code of Practice (Version 8) and gave informed consent. Protocols were approved by the University Ethical Committee (University of Birmingham Life and Health Sciences ERC 07-009). Semen collected by masturbation after 2–3 days of sexual abstinence was allowed to liquefy for ≈30 min (37°C, 6% CO_2_). Cells were harvested by direct swim-up into supplemented Earle's balanced salt solution (sEBSS), as described previously (Harper *et al.*, 2003) and adjusted to 6 × 10^6^ cells/ml. Aliquots of 200 μl were left to capacitate (37°C, 6% CO_2_) for 4–5 h.

#### Salines

sEBSS contained (mM) 118.4 NaCl, 5.4 KCl, 0.81 MgSO_4_·7H_2_O, 25.0 NaHCO_3_, 1.02 NaH_2_PO_4_, 5.5 C_6_H_12_O6, 2.5 C_3_H_3_NaO_3_, 19.0 CH_3_CH(OH)COONa, 1.8 CaCl_2_·2H_2_O and 15 HEPES (pH 7.35, 285–295 mOsm), supplemented with 0.3% (w/v) fatty acid-free bovine serum albumin (BSA).

Initial experiments with complete sEBSS showed no effect of the peptides used here, but when peptides were applied in BSA-free medium, clear effects of peptide treatment occurred. We therefore completed preparation of spermatozoa and transfer to the recording chamber in complete medium, but the cells were then superfused with BSA-free EBSS for ∼5 min before recording commenced. No peptide controls were run under similar conditions.

### Microwave-enhanced synthesis of proteomimetic bioportides

A polycationic sequence derived from human STIM (STIM^371-392^; KQLLVAKEGAEKIKKKRNTLFG) [KIKKK] and a scrambled homologue (LKNKFKGVKLAEIEKQALKGTR) [scrambled KIKKK] were synthesized using microwave-assisted solid-phase peptide synthesis. Syntheses (0.1 mmol scale) were performed using a Discover SPS Microwave Peptide Synthesizer (CEM Microwave Technology Ltd, Buckingham, UK) with fibre optic temperature control employing an N-α-Fmoc protection strategy with *O*-(1H-6-Chlorobenzotriazole-1-yl)-1,1,3,3-tetramethyluronium hexafluorophosphate (HCTU) activation on Rink amide methylbenzhydrylamine resins pre-loaded with the first amino acid (AA; AnaSpec, Inc., Cambridge Bioscience Ltd, Cambridge, UK). Deprotection with 7 ml of 20% piperidine was performed for 3 min at 50 W/75°C. A majority of AA coupling reactions were accomplished with a 4-fold molar excess of Fmoc-protected AA with HCTU and diisopropylethylamine (DIPEA), molar ratio of 1 : 1 : 2 (AA/HCTU/DIPEA), in 4 ml for 10 min at 25 W/75°C. Arg coupling was performed in two stages: 30 min 0 W/∼25°C followed by 5 min at 17 W/75°C. N-terminal acylation with 6-carboxy-tetramethylrhodamine (TAMRA; Novabiochem, Beeston, UK) yielded fluorescent peptides to confirm efficient uptake efficacy (Jones *et al.*, 2013).

Crude peptides were purified to apparent homogeneity using semipreparative scale, reversed-phase high-performance liquid chromatography. The predicted masses of all peptides used (average M+H^+^) were confirmed to an accuracy of +1 by matrix-assisted laser desorption/ionization time-of-flight mass spectrometry (Jones *et al.*, 2008). The ability of KIKKK and scrambled KIKKK to penetrate sperm was initially tested using TAMRA-labelled versions of the peptides. KIKKK accumulation appeared to exceed that of the scrambled peptide (Supplementary Fig. S1), probably reflecting specific binding of KIKKK after membrane translocation.

### Single-cell imaging of [Ca^2+^]_i_

Loading of cells with Oregon Green BAPTA 1 (Invitrogen) and time-lapse fluorescence imaging was done as described previously (Nash *et al.*, 2010). All experiments were performed at 25 ± 0.5°C in a continuous flow of medium (sEBSS). Images were captured at 0.1 Hz using a ×40 oil immersion objective and a Q Imaging Rolera-XR cooled CCD camera or Andor Ixon 897 EMCCD camera controlled by iQ software (Andor Technology, Belfast, UK; Nash *et al.*, 2010).

KIKKK and scrambled KIKKK were applied by addition to the perfusion header at 5 µM, a concentration that provides optimal loading of mammalian spermatozoa with CPP within minutes without compromising membrane integrity/cell viability and distinguishes clearly between peptides with high and low translocation efficiency (Jones *et al.*, 2013).

### Data analysis

Analysis of images, background correction and normalization of data were performed using IQ (Andor; Northern Ireland) and Excel (Microsoft) as described previously. Unless stated otherwise, the region of interest was drawn around the posterior head and neck (PHN) region of each cell. Raw intensity values were imported into Microsoft Excel and normalized using the following equation:

$$\Delta F=\left[\frac{\left(F-F_{\mathrm{rest}}\right)}{F_{\mathrm{rest}}}\right]\times 100\%,$$

where Δ*F* is % change in intensity at time *t*, *F* is fluorescence intensity at time *t* and *F*_rest_ is the mean of ≥10 determinations of *F* during the control period. For comparing responses between experiments, we calculated and plotted *R*_tot_, the mean Δ*F* of all cells (*n* = 25–75) in the experiment.

Amplitude of the progesterone-induced [Ca^2+^]_i_ transient was calculated from the three points spanning the peak of the Δ*F*_mean_ trace. Amplitude at 3 min after progesterone application was calculated using six consecutive points spanning 2.5–3.5 min after application of progesterone.

To calculate the proportion of cells giving a significant response to stimulation, we used the mean and 95% confidence intervals of the 10 data points immediately preceding stimulation and the three points spanning the peak of change in fluorescence in the *R*_tot_ trace. The response is considered significant if the difference between control and response peak is greater than the sum of the two confidence intervals. Where one sample is much less variable than the other (which is the case when the control [Ca^2+^]_i_ signal is both stable and ‘quiet’), then alpha approaches 0.05. If both samples show variability (as when the control [Ca^2+^]_i_ signal is ‘noisy’ or if the fluorescence intensity is drifting), the test becomes more stringent, the alpha level falling to ≈0.008 when the SEM of the two samples is equal.

Values given in the results and in figures are shown as ±SEM. For testing significance, a value of *P* < 0.05 was considered significant. Calculation of confidence intervals, SEM and testing of statistical significance was carried out using Excel (Microsoft).

Progesterone and all materials for salines were from Sigma, Poole, UK, except BSA which was from Stratech Scientific, Newmarket, UK.

## Results

**Figure 1:**
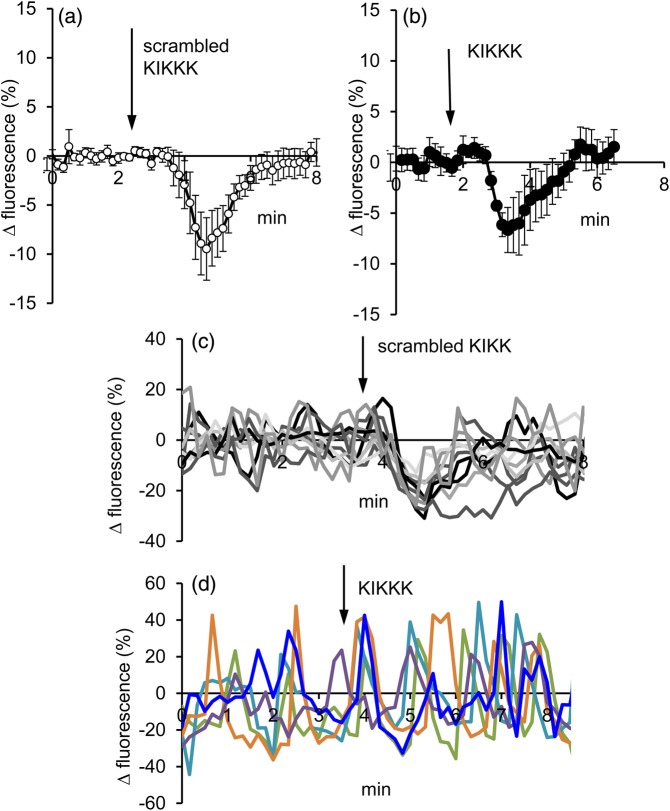
Effect of cell-penetrating peptides on resting [Ca^2+^]_i_ in human sperm. In this, and subsequent figures, data are expressed as % change in fluorescence. (**a**) Effect of 5 µM scrambled KIKK peptide, and (**b**) effect of 5 µM KIKK peptide. Arrows indicate time of addition of peptide. Traces show mean (±SEM) *R*_tot_ for eight experiments. (**c**) Single-cell traces showing transient fall in [Ca^2+^]_i_ upon application of KIKKK. (**d**) Effect of KIKKK in cells showing spontaneous [Ca^2+^]_i_ oscillations. In a few cells, oscillations ‘paused’ briefly (blue trace) but in the majority they persisted and kinetics appeared unchanged.

We have shown previously that CPPs enter mammalian spermatozoa rapidly, with a half-time between 1 and 3 min, dependent on the CPP employed (Jones *et al.*, 2013). Therefore, rather than comparing separate groups of treated and non-treated cells, the effects of CPPs on resting [Ca^2+^]_i_ was monitored during application. Exposure of human sperm to 5 µM KIKKK induced a transient decrease in [Ca^2+^]_i_ that was detectable within <30 s, was maximal at ≈90 s (mean decrease in *R*_tot_ of 6.6 ± 3.2%; *n* = 8 experiments; *P* < 0.05 compared with pre-stimulation) and recovered to control level within ∼3–4 min (Fig. 1). Application of scrambled KIKKK induced a similar effect (*P* < 0.025 versus pre-stimulation; Fig. 1). The amplitude of this effect varied between cells (Fig. 1c), but both KIKKK and scrambled KIKKK induced a significant decrease in fluorescence (see Methods) in ≈65% of cells. Twenty percent of cells (19.2 ± 2.7%; *n* = 18 experiments) were spontaneously active, generating [Ca^2+^]_i_ oscillations under control conditions. In ∼40% of these cells, application of CPPs briefly arrested and reset this activity (KIKKK = 42.8 ± 7.8%, *n* = 9 experiments; scrambled KIKKK = 39.8 ± 9.0%, *n* = 9 experiments; *t* > 0.8), but oscillation amplitude and kinetics were maintained, indicating that basal Ca^2+^ influx and Ca^2+^ clearance mechanisms were not affected by KIKKK or scrambled KIKKK peptide treatment (Fig. 1d).

**Figure 2:**
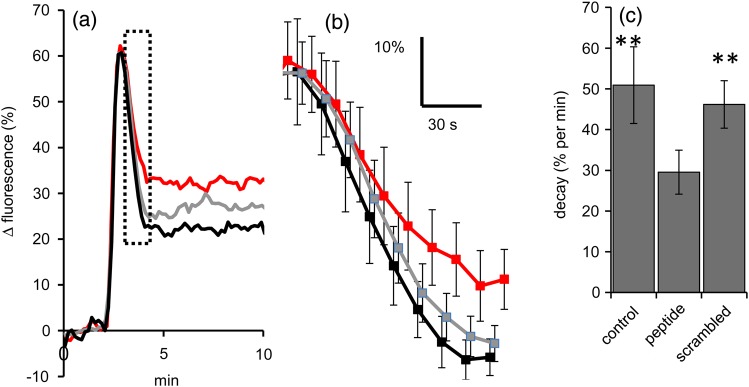
Pretreatment with KIKK prolongs the progesterone-induced [Ca^2+^]_i_ transient in human sperm. (**a**) Effects of 5 µM KIKKK peptide (red) and 5 µM scrambled KIKKK peptide (grey) on progesterone-induced biphasic [Ca^2+^]_i_ response in human sperm. Black shows response to progesterone in cells with no pretreatment. Each trace is the mean of *R*_tot_ plots (average of all cells in an experiment) from nine experiments (±SEM). Arrows indicate time of addition of progesterone. (**b**) Enlarged plot of boxed section from (a) with error bars (±SEM). (**c**) Mean rate of decay of *R*_tot_ (from 1.5 to 2 min after application of progesterone; mean ± SEM from nine sets of parallel experiments). **P < 0.01 compared with KIKKK pretreatment (paired *t*-test).

We then investigated whether KKKK could affect Ca^2+^ signals induced by the CatSper agonist progesterone. Experiments were carried out as ‘parallel’ sets, including a control to assess the effect of progesterone with no pretreatment and experiments using pretreatment with KIKKK and with scrambled KIKKK peptide. Stimulation of human sperm with 3 μM progesterone induces a biphasic [Ca^2+^]i response, comprising an early [Ca^2+^]_i_ transient which decays within 2 min, followed by a slowly developing sustained component (Blackmore *et al.*, 1990; Kirkman-Brown *et al.*, 2000). This biphasic response was clearly visible in *R*_tot_ plots (mean normalized responses of all cells in an experiment) of control experiments (Fig. 2a). Exposure to 5 μM KIKKK CPP prior to stimulation with 3 μM progesterone did not affect the mean amplitude or rise time of the [Ca^2+^]_i_ transient in *R*_tot_ traces (*P* > 0.75 compared with parallel controls and cells pretreated with scrambled peptide; Fig. 2a). However, in KIKKK-pretreated cells, the decay of the transient was clearly slower than in control experiments (untreated and pretreated with scrambled peptide). During the steepest part of the falling phase of the [Ca^2+^]_i_ transient (from 1.5 to 2 min after progesterone application), the rate of decay was significantly reduced in KIKKK-pretreatment experiments compared with parallel controls (no pretreatment and scrambled peptide pretreatment; Fig. 2c), such that in 8 of 9 experiments the amplitude of the early sustained response (*R*_tot_ assessed 2.5–3.3 min after application of progesterone) was significantly greater in cells pretreated with KIKKK (Fig. 2b; *P* < 0.03; paired *t*-test). This difference between control and pretreated cells persisted into the plateau phase of the [Ca^2+^]_i_ signal in some sets of experiments, but this was inconsistent and at 7.5 min after progesterone addition the three conditions did not differ statistically (*P* > 0.2).

**Figure 3:**
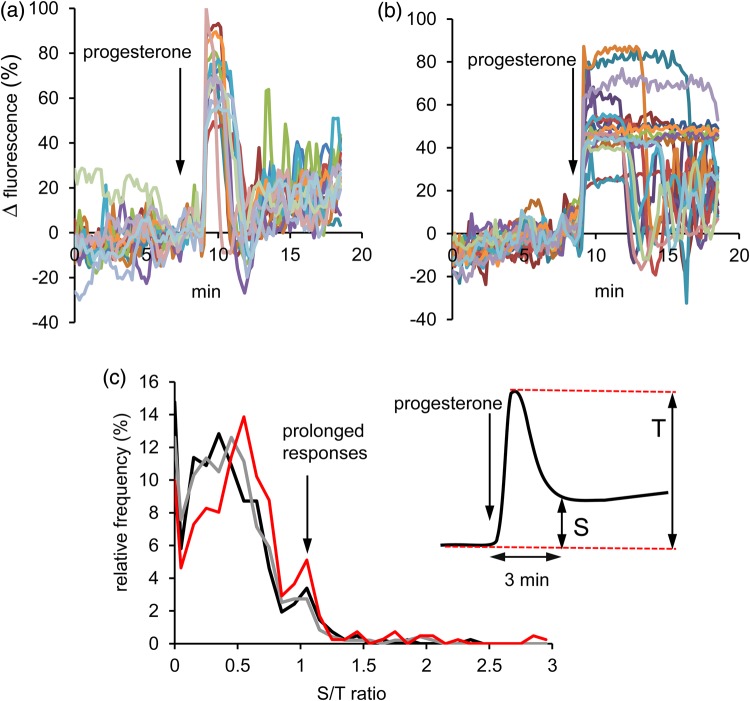
KIKKK pretreatment enhances the occurrence of prolonged progesterone-activated [Ca^2+^]_i_ transients in human sperm. (**a** and **b**) Single-cell [Ca^2+^]_i_ responses from the same (5 µM KIKKK pretreated) experiment which have been sorted into two populations according to whether the [Ca^2+^]_i_ transient starts to decay with 3 min of progesterone application [‘conventional’ transient; (a)] or persists at maximum for at least 3 min after progesterone application [prolonged response (b)]. (**c**) Distribution of the ratio of S/T ratios ([Ca^2+^]_i_ response amplitude at 3 min after progesterone application: amplitude of [Ca^2+^]_i_ transient peak—see inset). Black trace shows control, red trace shows cells pretreated with 5 µM KIKKK and grey trace shows cells pretreated with 5 µM scrambled KIKK. KIKKK pretreatment enhances the size of the modal S/T ratio and also increases the size of the shoulder at 0.9–1.1, which shows cells where the transient is maintained as a plateau for ≥3 min.

Though [Ca^2+^]_i_ responses to progesterone occur in ≈98% of human sperm (Kirkman-Brown *et al.*, 2000; Harper *et al.*, 2003), the response of the cells, as assessed by the amplitude and ‘shape’ of the [Ca^2+^]_i_ signal, can vary greatly within a sample (Harper *et al.*, 2003; Kirkman-Brown *et al.*, 2003). Pretreatment with 5 µM KIKKK slightly reduced the proportion of cells that generated a significant [Ca^2+^]_i_ response to progesterone compared with controls (mean control = 98.5 ± 1.0%; KIKK = 95.9 ± 1.3%; *n* = 9, *P* < 0.05, paired *t*-test on arcsine transformed data), a similar effect occurring with scrambled KIKK (96.1 ± 1.3%; *n* = 9; *P* > 0.7 compared with KIKKK). However, inspection of single-cell traces showed that there was a marked increase in a subpopulation of ‘atypical’ responses where the decay of [Ca^2+^]_i_ transient was greatly delayed, such that the [Ca^2+^]_i_ response appeared monophasic rather than the normal biphasic pattern (Fig. 3a and b). Assessment of the frequency of prolonged responses by visual inspection (defined as no discernible decay of the [Ca^2+^]_i_ signal for ≥3 min after application of progesterone) gave values of 8.8% in control experiments with no pretreatment (424 cells; 9 experiments), 6.3% after pretreatment with scrambled peptide (474 cells; 9 experiments; *P* = 0.20 compared with control; *χ*^2^) and 15.2% after pretreatment with KIKKK peptide (408 cells; 9 experiments; *P* = 1.9 × 10^−7^ compared with scrambled; *χ*^2^). To further quantify the occurrence of prolonged [Ca^2+^]_i_ transients in KIKKK-pretreated cells, we calculated for each cell the mean amplitude (normalized fluorescence) of the early sustained [Ca^2+^]_i_ signal for the period from 3 to 5 min after progesterone application (immediately after termination of a normal [Ca^2+^]_i_ transient) and normalized this value to the amplitude of the preceding transient in that cell (S/T ratio). For control cells, the S/T ratio was 0.36 ± 0.03 (424 cells, 9 experiments) and this was not significantly altered in cells pretreated with scrambled peptide (0.39 ± 0.06; 474 cells, 9 experiments; *P* = 0.63). However, in cells pretreated with KIKKK, this ratio was significantly increased (*P* < 0.003) to 0.55 ± 0.05 (408 cells, 9 experiments). The frequency distribution of S/T ratios was clearly bi-modal. In control and scrambled peptide pretreated cells, most S/T ratios formed a peak with a mode of ≈0.4, but there was also a clear shoulder at 1.0, reflecting cells in which the initial [Ca^2+^]_i_ ‘transient’ had not decayed within 3 min (Fig. 3d). In KIKKK-pretreated cells, the ratios were higher (mode increased from ≈0.4 to 0.55) and the occurrence of ratios >0.9 was significantly increased (*P* < 0.05; *χ*^2^). We conclude that pretreatment with KIKKK increases the proportion of cells in which the progesterone-induced [Ca^2+^]_i_ transient persists for several minutes before decaying.

**Figure 4:**
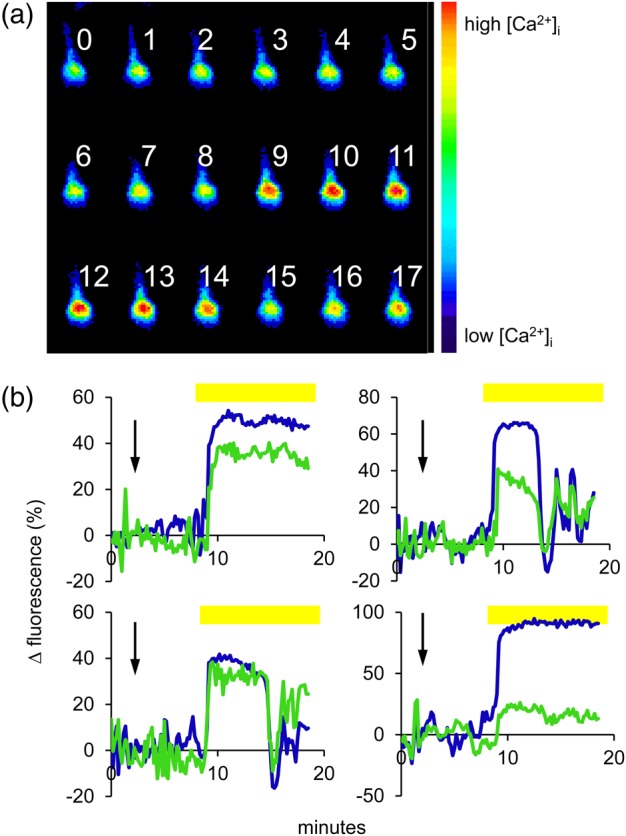
Prolonged [Ca^2+^]_i_ responses in human sperm are not due to mitochondrial Ca^2+^ accumulation. (**a**) Montage shows a pseudocolour image series of a 5-µM KIKKK-pretreated cell which shows a prolonged [Ca^2+^]_i_ response. Images are at 1 min intervals (indicated by adjacent numbers). Progesterone was added just before image 9. Note that increased [Ca^2+^]_i_ at the posterior head/neck (PHN) is maintained for >4 min. (**b**) Four examples showing separate analysis of [Ca^2+^]_i_ responses in the midpiece (green) and PHN region (blue) in the same cell. KIKKK was added at the arrow; yellow bars show application of 3 µM progesterone. Responses occur simultaneously the PHN and midpiece.

In our previous work on the effects of 2-APB on the progesterone-induced [Ca^2+^]_i_ signal, we observed strong enhancement of the sustained Ca^2+^ response that was localized specifically to the midpiece, possibly reflecting enhanced accumulation of Ca^2+^ in the mitochondria due to the effects of the drug on mitochondrial Ca^2+^ export (Lefievre *et al.*, 2012). To assess whether the effect described here might be similar, we investigated the location of the [Ca^2+^]_i_ signal. Prolonged responses occurred simultaneously in the midpiece and PHN of the sperm, showing that this effect was primarily on cytoplasmic [Ca^2+^]_i_ and not due to Ca^2+^ accumulation within the mitochondrial matrix (Fig. 4).

## Discussion

We showed previously, using fluorescently tagged peptides, that CPPs readily enter mammalian sperm, reaching 50% of their final intracellular concentration within 1–3 min (Jones *et al.*, 2013). In the present study, when KIKKK peptide was applied to human sperm, we observed a fall in [Ca^2+^]_i_ which recovered to control levels within 3–4 min, often causing a ‘resetting’ of activity in spontaneously oscillating cells. The kinetics of this effect are consistent with rapid entry of the peptide into human sperm but, since this effect was also seen upon application of scrambled KIKKK, it clearly does not reflect a specific effect of the KIKKK sequence on regulation of SOCs. One intriguing possibility is that, since both KIKKK and scrambled peptides bear a net positive charge (7 basic residues out of a total of 22), it binds electrostatically to the net negative charge on the membrane surface. Until equilibration of the peptide across the membrane is reached, this binding is asymmetric, making the extracellular surface more positive and increasing the voltage gradient within the membrane. This effect, termed charge screening, functionally hyperpolarizes the membrane and can reduce the open probability of voltage-sensitive channels (such as CatSper) sufficiently to inhibit passive Ca^2+^ influx and lower resting [Ca^2+^]_i_ (Wilson *et al.*, 1983). As the peptide accumulates within the sperm, the asymmetry of this effect will decay, allowing passive Ca^2+^ influx and [Ca^2+^]_i_ to return to resting levels.

Stimulation of human sperm with nM–µM concentrations of progesterone elicits an immediate [Ca^2+^]i transient that is dependent on Ca^2+^ influx through CatSper channels (Strunker *et al.*, 2011). Under the conditions of our imaging experiments, [Ca^2+^]_i_ peaks in 15–30 s and decays over 1–2 min (Kirkman-Brown *et al.*, 2000; Harper *et al.*, 2003). In populations of sperm pretreated with the KIKKK peptide, there was no change in the rise time or amplitude of this [Ca^2+^]_i_ transient but decay was slower, such that 3 min after progesterone stimulation the [Ca^2+^]_i_ in pretreated cells was ≈1.5-fold higher than in control cells or cells pretreated with the scrambled peptide. Analysis of single-cell records showed that this effect was not due to a reduced decay rate of Ca^2+^ clearance, but reflected an increase in the proportion of cells in which the transient peak was abnormally prolonged into a plateau. These monophasic responses were not restricted to the midpiece, occurring simultaneously at the PHN, and thus reflect increased cytoplasmic [Ca^2+^]_i_ rather than Ca^2+^ accumulation in the mitochondria (Fig. 4). The slow decay of the sperm population [Ca^2+^]_i_ signal results from cell–cell variation in the duration of these abnormal [Ca^2+^]_i_ transients. Interestingly, such prolonged transients also occur in untreated cells, but their incidence was greatly enhanced by pretreatment with KIKKK.

These observations suggest that SOCs at the sperm neck (Lefievre *et al.*, 2012) become activated and contribute a second Ca^2+^-influx pathway. A number of strands of evidence point to rapid recruitment of such a secondary Ca^2+^ signal downstream of CatSper-mediated Ca^2+^ influx in human sperm:

- Low-dose (5 µM) 2-APB, which sensitizes SOC activation but does not affect CatSper currents, enhanced [Ca^2+^]_i_ elevation during the initial progesterone-induced transient. This effect was localized to the sperm neck, but did not occur in the flagellum (Lefievre *et al.*, 2012).
- Treatment with 1–10 µM nifedipine significantly reduces the duration (but not amplitude) of the progesterone-induced [Ca^2+^]_i_ transient, apparently inhibiting a ‘late’ component of the transient which activates 10–20 s after initiation of the response (Kirkman-Brown *et al.*, 2003). Since 10 µM nifedipine did not inhibit CatSper (Strunker *et al.*, 2011) and there is no evidence for activation of nifedipine-sensitive voltage-activated Ca^2+^ channels by progesterone, we consider it likely that this action is exerted on Ca^2+^ store mobilization or on SOCs.
- In progesterone-treated sperm, [Ca^2+^]_i_ oscillations, consistent with cyclical mobilization of stored Ca^2+^, occur after the initial [Ca^2+^]_i_ transient. These are dependent on a low level of Ca^2+^-influx and are sensitive to manipulation of ryanodine receptors (Harper *et al.*, 2004; Kirkman-Brown *et al.*, 2004; Aitken and McLaughlin, 2007; Sanchez-Cardenas *et al.*, 2014).

Since Ca^2+^ stores, STIM and Orai proteins are present at the sperm neck, the simplest interpretation consistent with these observations is that CatSper-mediated Ca^2+^-influx into the flagellum induces secondary mobilization of stored Ca^2+^ and activation of CCE. Upon decay of the CatSper-mediated Ca^2+^ influx, Ca^2+^ stores will refill and CCE will also decay, dependent on successful termination of STIM–Orai interaction, such that interference with the regulatory mechanism on STIM delays (though does not prevent) decay of the [Ca^2+^]_i_. If this model is correct, pharmacological blockade of SOCs should have effects on the progesterone-induced [Ca^2+^]_i_ signal. Indeed at high doses of 2-APB [100 µM, which inhibits SOC function (Lis *et al.*, 2007; DeHaven *et al.*, 2008)], the enhancement of the progesterone-induced [Ca^2+^]_i_ transient seen at low doses is reversed, the sustained component of the signal is suppressed and in some preparations the transient duration is reduced compared with parallel controls (Lefievre *et al.*, 2012). In experiments with the non-specific SOC blocker SKF 96365 (10–30 µM), we observed a rise in [Ca^2+^]_i_ in most cells, an effect which has been described previously (Jan *et al.*, 1999). When progesterone was subsequently applied, the sustained component was strongly inhibited and the duration (but not amplitude) of the progesterone-induced [Ca^2+^]_i_ transient was reduced compared with a parallel control experiment (L. Lefievre, J. Morris, S. Publicover; unpublished data). Though the effects of these drugs on the response to progesterone are consistent with activation of SOCs, the effects of SKF 96365 alone are such that they cannot be interpreted.

A peculiarity of SOCs in sperm is that, whereas in most cells STIM is distant from the plasmalemma and must be redistributed to interact with Orai, intracellular membranes of sperm are located close to the plasma membrane, such that no redistribution of STIM is discernible in cells that have not been stimulated to mobilize stored Ca^2+^ (Lefievre *et al.*, 2012). Thus, if the auto-inhibitory interactions in STIM are disrupted, activated SOCs may switch off relatively slowly even if store refilling occurs, leading to prolonged Ca^2+^ influx.

[Ca^2+^]_i_ signalling in human sperm is crucial in the regulation of a range of functions (Publicover *et al.*, 2007; Darszon *et al.*, 2011). Whereas previous work using pharmacological and immunological techniques suggested that a range of channels contributed to sperm Ca^2+^ signalling, data obtained from the application of patch clamp have revealed only currents carried by CatSper channels. The reason for this discrepancy is not clear, though whole-cell access and consequent cytoplasmic dialysis of the minute cytoplasmic volume might affect the functioning of some sperm ion channels (Publicover and Barratt, 2012). Evidence for participation of SOCs must therefore be interpreted cautiously, but the data are consistent with a flexible Ca^2+^-signalling system in which activation of SOCs can contribute a spatially and temporally separate route for influx of Ca^2+^ into the sperm, which may contribute to the ability of the cell to regulate separately several Ca^2+^-controlled processes (Alasmari *et al.*, 2013).

## Supplementary data

Supplementary data are available at http://molehr.oxfordjournals.org/.

## Authors’ roles

J.M., S.J., M.L. and L.L. carried out the experimental work and analysis of data; S.J.P. and J.H. designed the study and wrote the manuscript

## Funding

L.L. was supported by the Wellcome Trust (Grant #086470). J.M. was supported by a University of Birmingham Teaching Assistantship. Funding to pay the Open Access publication charges for this article was provided by the Welcome Trust.

## Conflict of interest

There is no conflict of interest that could be perceived as prejudicing the impartiality of the research reported.

## Supplementary Material

Supplementary Data
