# Supplementary material for: Cell-penetrating peptides, targeting the regulation of store-operated channels, slow decay of the progesterone-induced [Ca2+]i signal in human sperm
Source: Mol Hum Reprod. 2015 Apr 16;21(7):563–70. doi: 10.1093/molehr/gav019 (PMC4487447; doi:10.1093/molehr/gav019)
Supplement: Supplementary Data [file supp_21_7_563__index.html]

Cell-penetrating peptides, targeting the regulation of store-operated channels, slow decay of the progesterone-induced [Ca2+]i signal in human sperm — Cell-penetrating peptides, targeting the regulation of store-operated channels, slow decay of the progesterone-induced [Ca2+]i signal in human sperm — Supplementary Data 

# Cell-penetrating peptides, targeting the regulation of store-operated channels, slow decay of the progesterone-induced [Ca2+]i signal in human sperm

## Supplementary Data

Supplementary Data

**Files in this Data Supplement:**

- Supplementary Data - Docx file
