# Supplementary material for: Cell-penetrating peptides, targeting the regulation of store-operated channels, slow decay of the progesterone-induced [Ca2+]i signal in human sperm
Source: Mol Hum Reprod. 2015 Apr 16;21(7):563–70. doi: 10.1093/molehr/gav019 (PMC4487447; doi:10.1093/molehr/gav019)
Supplement: Supplementary Data [file supp_gav019_gav019supp.docx]

**Quantitative analysis of peptide translocation into bovine spermatozoa**. a: Bovine spermatozoa were incubated with 5 μM TAMRA-labelled CPP (Tat [(GRKKRRQRRRPPQ) - an inert CPP vector control], KIKKK and scrambled KIKKK) for 1 h at 37^o^C. Data are mean + S.E.M from 3 experiments. b: Uptake of KIKKK into bovine sperm. C: Uptake of scrambled KIKKK into bovine sperm. In both assays membrane bound peptide was removed by extensive washing and trypsin treatment.
